# Bradycardia unresponded to atropin testing was successfully treated with therapeutic plasma exchange in a patient with severe COVID-19 complicated by Guillain-Barré syndrome: A case report

**DOI:** 10.3389/fcvm.2022.1035896

**Published:** 2023-01-19

**Authors:** Sy Duong-Quy, Duc Huynh-Truong-Anh, Quynh Tran-Xuan, Tien Nguyen-Quang, Thanh Nguyen-Thi-Kim, Thanh Nguyen-Chi, Thuy Tran-Ngoc-Anh, Nam Nguyen-Van-Hoai, Mai Do-Thi-Thu, Tram Tang-Thi-Thao, Khue Bui-Diem, Tien Hoang-Anh, Thu Nguyen-Ngoc-Phuong, Vinh Nguyen-Nhu

**Affiliations:** ^1^Biomedical Research Center, Lam Dong Medical College, Da Lat, Vietnam; ^2^COVID-19 Unit of Phu Chanh, Binh Duong General Hospital, Thu° Dãu Môt, Binh Duong, Vietnam; ^3^Division of Pulmonary, Allergy, and Critical Care Medicine, Penn State College of Medicine, Hershey, PA, United States; ^4^Faculty of Medicine, Pham Ngoc Thach University of Medicine, Ho Chi Minh City, Vietnam; ^5^Department of Internal Medicine, Can Tho University of Medicine and Pharmacy, Can Tho, Vietnam; ^6^Department of Physiology and Pathophysiology, University of Medicine and Pharmacy at Ho Chi Minh City, Ho Chi Minh City, Vietnam; ^7^Department of Cardiology. Hue University of Medicine and Pharmacy, Hue University, Hue, Vietnam; ^8^Department of Family Medicine, Faculty of Medicine, University of Medicine and Pharmacy at Ho Chi Minh City, Ho Chi Minh City, Vietnam; ^9^Department of Respiratory Functional Exploration, University Medical Center, Ho Chi Minh City, Vietnam

**Keywords:** SARS-CoV-2, COVID-19, bradycardia, Guillain-Barrée syndrome, therapeutic plasma exchange

## Abstract

The severe acute respiratory syndrome coronavirus 2 (SARS-CoV-2) pandemic has been an alarming situation worldwide for the past 2 years. The symptoms of coronavirus disease 2019 (COVID-19) are not only confined to the respiratory system but also affect a multitude of organ systems. Bradycardia associated with Guillain-Barré syndrome (GBS) is a rare autonomic and peripheral neurological complication of COVID-19. In this case report, we present the case of a 26-year-old man diagnosed with bradycardia associated with GBS after contracting COVID-19. Initially, this patient had the classical symptoms of COVID-19 and was hospitalized in the intensive care unit (ICU) for acute respiratory distress syndrome (ARDS). Then, he developed weakness in the lower extremities, diminished tendon reflexes, a loss of sensation without sphincter muscle disorders, and bradycardia. His bradycardia did not respond to atropine. The patient was treated concurrently with a high-flow nasal cannula, systemic corticosteroids, anticoagulation, and therapeutic plasma exchange (TPE) for COVID-19-induced ARDS, bradycardia, and GBS. His ARDS and bradycardia improved after the first cycle of TPE and medical treatment. After three cycles of TPE, the patient progressively recovered his muscle strength in the lower limbs and regained peripheral sensation. He was discharged from the hospital in stable condition after 4 weeks of hospitalization and was followed up after 6 months for cardiorespiratory and neurological complications. This case report elucidates the potential difficulties and challenges that physicians may encounter in diagnosing and treating COVID-19-induced bradycardia and GBS during the pandemic outbreak. However, the patient outcomes with the treatment combining the conventional treatment with therapeutic plasma exchange seem to be optimistic.

## Introduction

The coronavirus disease 2019 (COVID-19) is caused by a coronavirus called severe acute respiratory syndrome coronavirus 2 (SARS-CoV-2), which has affected more than 600 million people worldwide. Severe COVID-19 infections have been significantly reduced due to the global efficacy of vaccination campaigns. A few days after contracting the virus, patients with COVID-19 commonly present with a wide range of symptoms, ranging from mild to severe. These symptoms include fever, cough, shortness of breath, sore throat, nausea, vomiting, and/or diarrhea. However, patients with advanced age or serious comorbidities remain at higher risk for developing serious complications from COVID-19.

However, patients with COVID-19 could present at an emergency department with diverse neurological symptoms, including confusion, refractory headache, new onset of anosmia and/or ageusia, and acute peripheral impairment. Although COVID-19-induced inflammatory polyradiculoneuropathy or Guillain-Barré syndrome (GBS) has been described previously (1–5), patients with COVID-19 complicated by bradycardia-associated GBS remain a rare cardio-neurological complication of COVID-19.

In this case report, we present the case of a young man with bradycardia-associated GBS after contracting COVID-19-induced acute respiratory distress syndrome (ARDS). The clinical and laboratory features of this patient were appropriate for the diagnosis of ARDS and bradycardia-associated GBS due to COVID-19. Although the bradycardia did not respond to atropine testing and the symptoms of GBS did not improve after the conventional treatment for acute COVID-19, the patient was successfully stabilized with a normal heart rate and stable cardiorespiratory status after therapeutic plasma exchange (TPE) in combination with standard treatment. This case suggests that TPE might be considered a useful approach to treatment when used in combination with conventional treatments for patients with severe cardiorespiratory and neurological complications of COVID-19.

## Case report

A 26-year-old man with a BMI of 34.6 kg/m^2^ was admitted to the Medical Center of Binh Duong Province, Vietnam in the middle of October 2021 because of fever, cough, vomiting, diarrhea, and shortness of breath. His laboratory test for COVID-19 from a nasal swab reverse transcription polymerase chain reaction (RT-PCR) test was positive within the cycle threshold of 16. He was previously in good health without any past medical history. He had already received two doses of the inactivated COVID-19 vaccine, the last of which he received 2 months before he was tested positive without any side effects. After his admission to the medical center, he was treated with oxygen therapy (12 L/min *via* Venturi mask), intravenous corticosteroids (dexamethasone 8 mg, two times a day), and anticoagulation (heparin).

He was transferred to the intensive care unit (ICU) of the COVID-19 Hospital of Phu Chanh, Binh Duong General Hospital on day 5 of his admission due to severe dyspnea and pain in his lower limbs associated with muscular weakness. At the time of his ICU arrival, he was conscious; his blood pressure was 130/70 mmHg; his heart rate was 75 beats/min; and his oxygen saturation (SpO_2_) was 90% with 15 L/min of oxygen through a mask. The neurological examination revealed the muscular weakness of the lower limbs with a Medical Research Council (MRC) scale of 3 out of 5 (vs. 5 out of 5 for the upper limbs), diminished sensation, and reflexes of the lower limbs without sphincter muscle disorders (Table 1). He had no neurogenic urinary or fecal incontinence.

**Table 1:** Laboratory data of the reported patient.

**Parameters**	**Upon admission to ICU**	**After day 14th**	**Institutional normal range**
RT-PCR SARS-CoV-2	+	Negative	Negative
White blood cell (10^9^/L)	8.4	11.1	4.0–11.0
*Neutrophil (%)*	74.11	66.89	45–75
*Lymphocyte (%)*	17.77	21.83	20–45
Platelet (10^9^/L)	351	246	140–500
CRP (mg/dL)	3.07	0.42	< 1.0
Lactate (mmol/L)	0.72	–	0.5–2.2
LDH (U/L)	405	306	< 247
Ferritin (ng/mL)	>1,500	347.4	23.9–336.2
Fibrinogen (g/L)	5.61	4.71	1.5–4.0
TP (%)	84	95	>70
APTT (s)	23.6	24.1	20–40
**Arterial blood gas**			
*pH*	7.32	7.32	7.35–7.45
*PaCO_2_ (mmHg)*	25	43	35–45
*HCO_3_^**−**^ (mmol/L)*	12.9	22.2	18–23
*Base Excess^**−**^ (mmol/L)*	−11.2	−4	−2–+3
*FiO2 (%)*	95	21	21
*PaO_2_ (mmHg)*	156	62	80–100
*A-aDO_2_*	490	169	5–20
PaO_2_/FiO_2_ (ratio)	164	295	400–500
Sodium (mmol/L)	131	132	135–145
Potassium (mmol/L)	4.1	**3.4**	3.5–5.0
Calcium (mmol/L)	1.2	1.1	1.1–1.6
Magnesium (mmol/L)	1.05	0.74	0.73–1.06
Urea (mmol/L)	14.2	2.4	2.8–7.2
Creatinine (μmol/L)	77	52	72–127
eGFR-MDRD (mL/m/m^2^)	113	126	≥ 60
HbA1c	12.5	6.2	< 5.7
AST (U/L)	34	22	0–50
ALT (U/L)	38	21	0–50
Glucose (mg/dL)	444	181	< 120
Total protein (g/L)	71	69	66–83
Albumin (g/L)	34	35	35–52
**Cerebrospinal fluid**			
*Cells*	0	–	0
*Protein (g/L)*	0.32	–	0.15–0.45
*Glucose (mmol/L)*	NA	–	NA
*Lactate (mmol/L)*	1.4	–	1.1–2.4
*Pandy's test*	negative	–	Negative

APTT, activated partial thromboplastin time; AST, aspartate aminotransferase; ALT, alanine aminotransferase; CRP, C-reactive protein; LDH, lactate dehydrogenase; eGFR, estimated glomerular filtration rate; FiO_2_, fraction of inspired oxygen; MDRD, Modification of Diet in Renal Disease; PaO_2_, partial pressure of oxygen in arterial blood; PaCO_2_, partial pressure of carbon dioxide in arterial blood; RT-PCR, reverse transcription polymerase chain reaction; SARS-CoV-2, severe acute respiratory syndrome coronavirus 2; TP, prothrombin time.

The chest x-ray showed diffuse ground-glass opacity. Blood analysis and biochemical laboratory tests confirmed increased glucose, fibrinogen, ferritin, and lactate dehydrogenase (LDH) levels. He had normal white blood cells, electrolyte parameters, and liver and kidney functions. His cerebrospinal fluid (CSF) was clear and colorless and without an elevation in white blood cells; the analysis of the CSF demonstrated albuminocytologic dissociation. The result of magnetic resonance imaging (MRI) of the spine was normal. COVID-19-induced ARDS associated with Guillain-Barré syndrome was diagnosed by hospital-qualified neurologists. Other diagnoses, such as compressive myelopathy, transverse myelitis, or acute myelitis, were excluded at that time.

The patient was treated with a high-flow nasal cannula (HFNC) with a fraction of inspired oxygen (FiO_2_) of 60%, an oxygen flow rate of 40 L/min, intravenous corticosteroids (dexamethasone: 8 mg, two times a day), anticoagulation (heparin), an intravenous antibiotic for nosocomial pneumonia prevention (ceftazidime and levofloxacin), and a subcutaneous insulin injection for hyperglycemia. The neurological symptoms worsened in the next 2 days, with an MRC scale of 1 out of 5 in the lower limbs and 2–3 out of 5 in the upper limbs, a decrease in the loss of superficial and deep sensation, and a decrease in reflexes. He did not have other autonomic symptoms such as blood pressure changes, bowel malfunctions, or urinary abnormalities. However, the patient then developed sinus bradycardia (40 beats/min), which did not respond to intravenous atropine.

**Figure 1:**
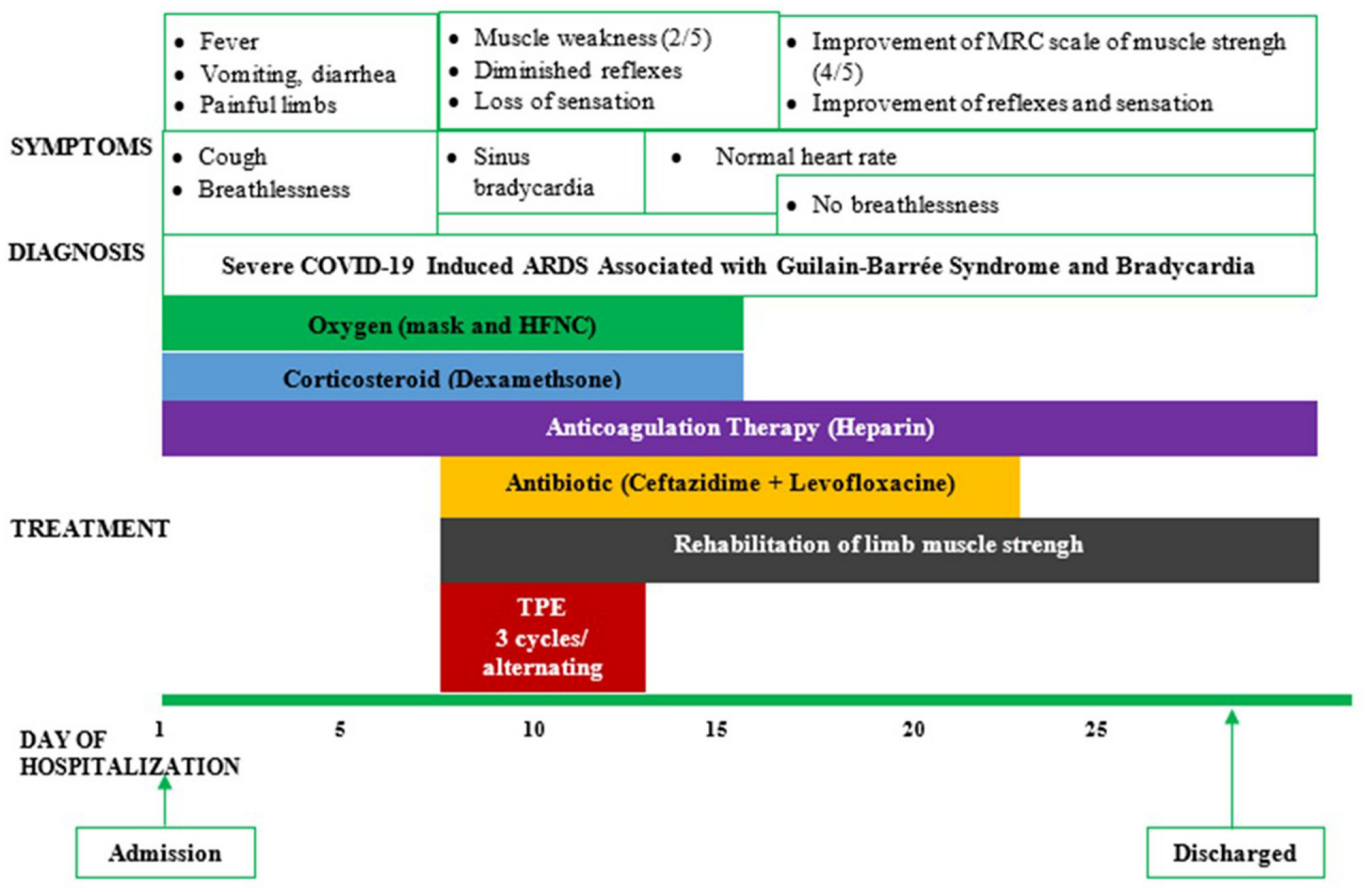
Symptoms, diagnosis, and treatment of a reported patient. ARDS, acute respiratory distress syndrome; HFNC, high-flow nasal cannula; TPE, therapeutic plasma exchange.

The patient was then treated with therapeutic plasma exchange (TPE) after the decision was made by hospital experts. TPE was done every 2 days with 5% human albumin replacement (4,700 ml/cycle). His bradycardia improved to a normal resting heart rate (>55 beats/min) after the first TPE. He did not experience dyspnea after 5 days of HFNC treatment with flow decreased from 40 to 15 L/min; he was then switched to a nasal cannula with 5 L/min, then 3 L/min, and finally stopped oxygen therapy on day 14 (Figure 1). The neurological symptoms of the patient also improved after day 10 with the progressive recovery of skin sensation and muscle tone in all four limbs evaluated by the MRC scale. The patient was discharged after 4 weeks of hospitalization and followed up regularly every 3 months until now. He recovered to his baseline health status after 3 months and returned to work without any health sequelae 6 months after discharge from the hospital. Currently, the patient is in good health with an MRC scale of 5 out of 5 in the lower and upper limbs and a normal resting heart rate. He has been seen regularly in the Outpatients Medical Center of Binh Duong General Hospital every 2–3 months.

## Discussion

In this case report, we present the case of a patient with severe COVID-19 associated with ascending symmetrical limb weakness, external sensation disturbances, absent deep tendon reflexes, and bradycardia. Especially, the CSF showed albuminocytologic dissociation, which supported the diagnosis of GBS in this patient. Thus, clinical examination results and laboratory data were appropriate for the diagnosis of severe COVID-19 complicated by GBS associated with bradycardia.

Initially, the patient presented with acute polyneuropathy after being diagnosed with COVID-19-induced pneumonia. Thus, the diagnosis of GBS seemed to be more accurate. Other diagnoses were eliminated, such as spinal cord compression from degenerative disc disease, spinal cord infarction, and transverse myelitis. During his illness, the patient had no neurogenic urinary, fecal incontinence, or any history of spinal trauma; therefore, the possibility of spinal cord compression was less likely. In addition, he did not experience sudden and severe back pain accompanied by numbness and tingling along the circumference of the spinal cord for diagnosing spinal cord infarction. It was also less likely to be acute transverse myelitis as the major motor findings of this condition are paraparesis or quadriparesis. In addition, the result of the spinal MRI did not show any abnormalities.

Our patient had bradycardia and his heart did not respond to atropine testing. Therefore, severe COVID-19 complicated by GBS associated with bradycardia was diagnosed by the local expert committee of COVID-19 Phu Chanh Hospital. Indeed, following one of the first cases of GBS-associated SARS-CoV-2 infection reported by Zhao et al. in 2020, numerous cases have been published during the COVID-19 pandemic (6–25). Arrhythmias were also one of the significant complications of COVID-19 reported in the world literature. In a recent study from Wuhan, China, cardiac arrhythmias developed in 16.7% of hospitalized patients and 44.4% of ICU patients with COVID-19 (26). In some case reports, patients developed sinus bradycardia related to COVID-19 despite routine echocardiography and cardiac biomarkers (27, 28).

Guillain-Barré syndrome and arrhythmias can develop after immunization or the SARS-CoV-2 infection. According to the safety committee of the European Medicine Agency, 108 cases of GBS were reported worldwide among over 21 million people who had received the vaccine as of 30 June 2021 (29). A 2021 study of Adverse Events Reported From COVID-19 Vaccine Trials based on the WHO database reported 4,863 cardiovascular adverse events, in which tachycardia was 16.41% and bradycardia was 5.2% (30). Our patient had his last vaccination dose 2 months before contracting COVID-19.

Autonomic neuropathy is a severe consequence of GBS. Overreactivity of vagal function, in particular, can cause major cardiac problems ranging from bradycardia to asystole (31). According to Mukerji et al., bradyarrhythmia can occur in up to 50% of patients with GBS (32). Cardiac arrhythmia was one of the adverse side effects observed during plasmapheresis procedures (33–35). Our patient recovered from sinus bradycardia after having the first TPE without recurrences during his hospital stay in the ICU. Therefore, this bradycardia could be considered a cardiac adverse event associated with GBS as a complication of COVID-19.

In this case report, TPE was used in combination with standard therapy, including oxygen therapy, anticoagulation, and corticosteroids, to treat severe COVID-19 (Figure 1). This combined treatment has demonstrated therapeutic effectiveness in our patient. Especially, the patient is currently in good health without any sequelae of GBS and post-COVID symptoms during his regular follow-up at our medical center. Although TPE, a therapeutic procedure used to eliminate antibodies and other potentially detrimental factors from the bloodstream (36), was the treatment option for patients with GBS before the COVID-19 pandemic, TPE may be used in COVID-19-induced GBS associated with bradycardia. In patients with severe COVID-19, TPE might be considered an efficient therapy for reducing the risk of cytokine storm-induced ARDS as it might reduce the inflammatory cytokines (including IL-6) and acute phase proteins (ferritin and CRP). This could help in improving tissue oxygenation and reducing the risk of hypercoagulation and improve tissue oxygenation and especially fatal cardiovascular events (37, 38).

Finally, the main limitation of this case report is the lack of other specific tests, such as electrophysiologic analysis, to confirm the diagnosis of GBS (39). It could not be performed on this patient due to the overcrowding of the local hospitals during the 4th wave of the COVID-19 pandemic in Vietnam (July–November 2021). Especially, at that moment, the mentioned ICU department held 125 patients with severe COVID-19 per day and more than half of them with mechanical ventilation, which induced the lack of medical staff for doing some specific tests in patients with COVID-19. Another possible limitation of this case report might be due to other potential causes of bradycardia that could not be eliminated in the reported patient (40–44).

## Conclusion

COVID-19 remains a critical health problem worldwide. Encouragingly, severe cases of COVID-19 have been significantly reduced after implementing national and international COVID-19 vaccination campaigns. This case report suggests that, in a patient with severe COVID-19 complicated by GBS associated with bradycardia, early treatment with therapeutic plasma exchange combined with standard therapy for COVID-19 may improve the outcome of the patient.

## Data availability statement

The raw data supporting the conclusions of this article will be made available by the authors, without undue reservation.

## Ethics statement

The studies involving human participants were reviewed and approved by Binh Duong General Hospital. The patients/participants provided their written informed consent to participate in this study.

## Author contributions

The literature search was done by SD-Q, DH-T-A, QT-X, TN-Q, TN-T-K, TN-C, and TT-N-A, with significant contributions from NN-V-H, MD-T-T, TT-T-T, KB-D, TH-A, TN-N-P, and VN-N. Data collection was done by SD-Q, DH-T-A, QT-X, TN-Q, TN-T-K, TN-C, TT-N-A, NN-V-H, MD-T-T, and TT-T-T. SD-Q, QT-X, and DH-T-A drafted the manuscript, with significant contributions by TN-Q, TN-T-K, TN-C, TT-N-A, NN-V-H, MD-T-T, TT-T-T, KB-D, TH-A, and TN-N-P. All authors contributed equally to analyzing and interpreting the data in the case report.
